# Planetary defense with the Double Asteroid Redirection Test (DART) mission and prospects

**DOI:** 10.1038/s41467-022-35561-2

**Published:** 2023-03-01

**Authors:** Andrew S. Rivkin, Andrew F. Cheng

**Affiliations:** Johns Hopkins University Applied Physics Laboratory, Laurel, MD USA

**Keywords:** Asteroids, comets and Kuiper belt, Astronomical instrumentation

## Abstract

NASA’s Double Asteroid Redirection Test (DART) mission intentionally impacted the asteroid Dimorphos on September 26, 2022, and this kinetic impact changed Dimorphos’ orbit around its binary companion Didymos. This first planetary defense test explored technological readiness for this method of asteroid deflection.

## DART mission

The history of life on Earth has been dramatically influenced by asteroid impacts. Tens of thousands of objects larger than 140 meters, capable of causing regional destruction, orbit the Sun in near-Earth orbits, but less than half have been found. A variety of mitigation techniques have been considered in case an incoming object is ever detected. One such technique, the kinetic impactor, is conceptually simple: A spacecraft is purposefully collided with the asteroid of concern, and the addition of the spacecraft momentum alters the asteroid orbit so that it no longer hits the Earth.

**Fig. 1:**
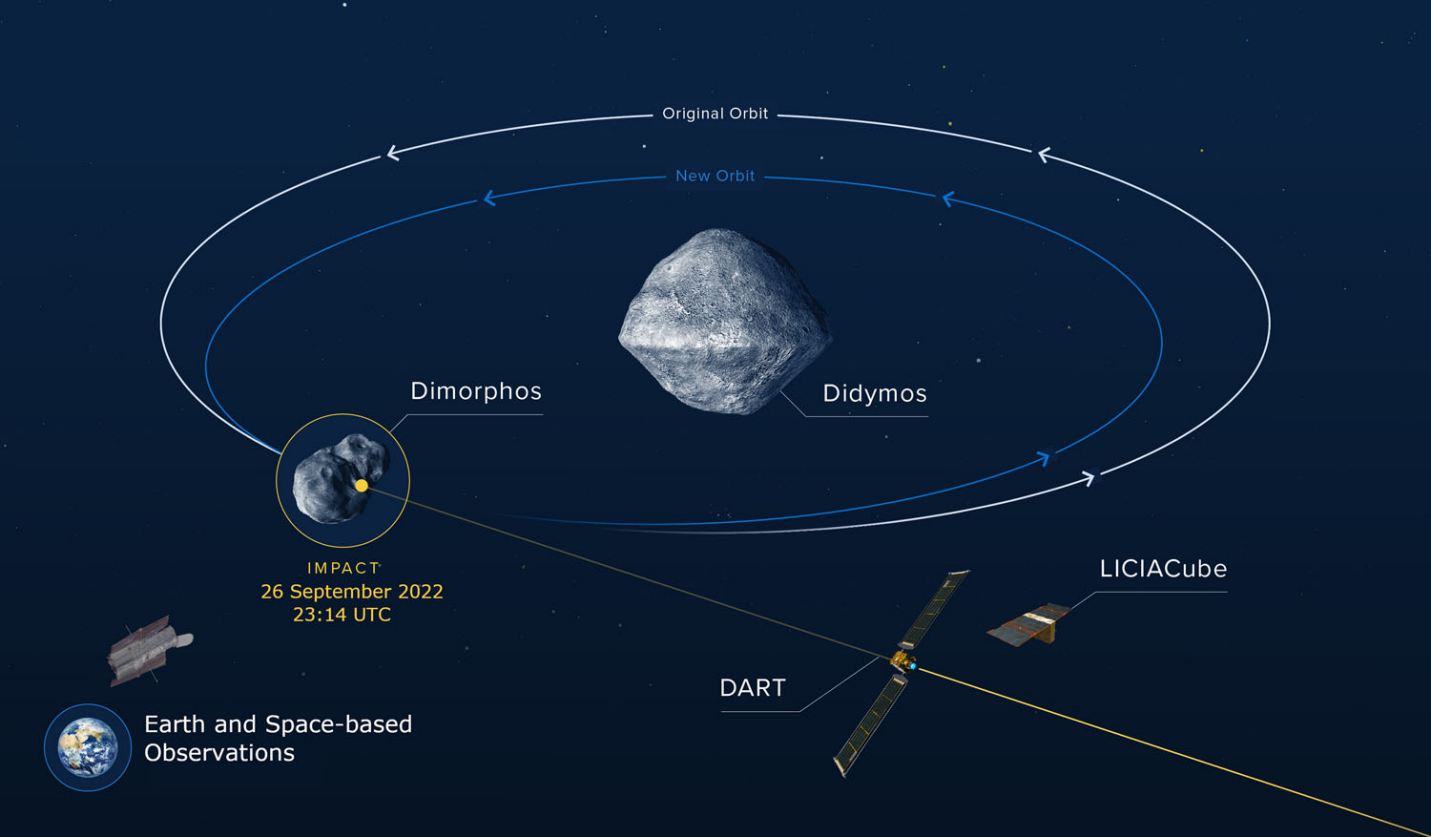
Schematic representation of the DART mission. The impact of the DART spacecraft into Dimorphos was observed by the LICIACube CubeSat and telescopes at the Earth and in space. As a result of the impact, Dimorphos’ orbit period was shortened and the mean distance from Dimorphos to Didymos was reduced. Credit: NASA/Johns Hopkins APL, adapted with permission.

The DART mission was launched in November 2021 as a demonstration of the kinetic impactor technique. DART was sent to the Didymos asteroid system, which contains the 750-m diameter Didymos and its 150-m moon Dimorphos and is not an impact hazard to Earth^1^. The purposeful impact shortened Dimorphos’ 12-h orbital period around Didymos by about half an hour^2,3^. This change is schematically demonstrated in Fig. 1. Images taken by DART showed Dimorphos to be a mixed collection of rocks from centimeters to tens of meters in size, jumbled together but making a remarkably smooth overall shape compared to other asteroids visited by spacecraft. Investigation of surface features (i.e. distribution of boulders, potential identification of craters, or evidence of structures like ridges or cracks) will provide clues about properties like Dimorphos’ density and subsurface layering, and will be used to help better interpret and calibrate the DART results.

**Fig. 2:**
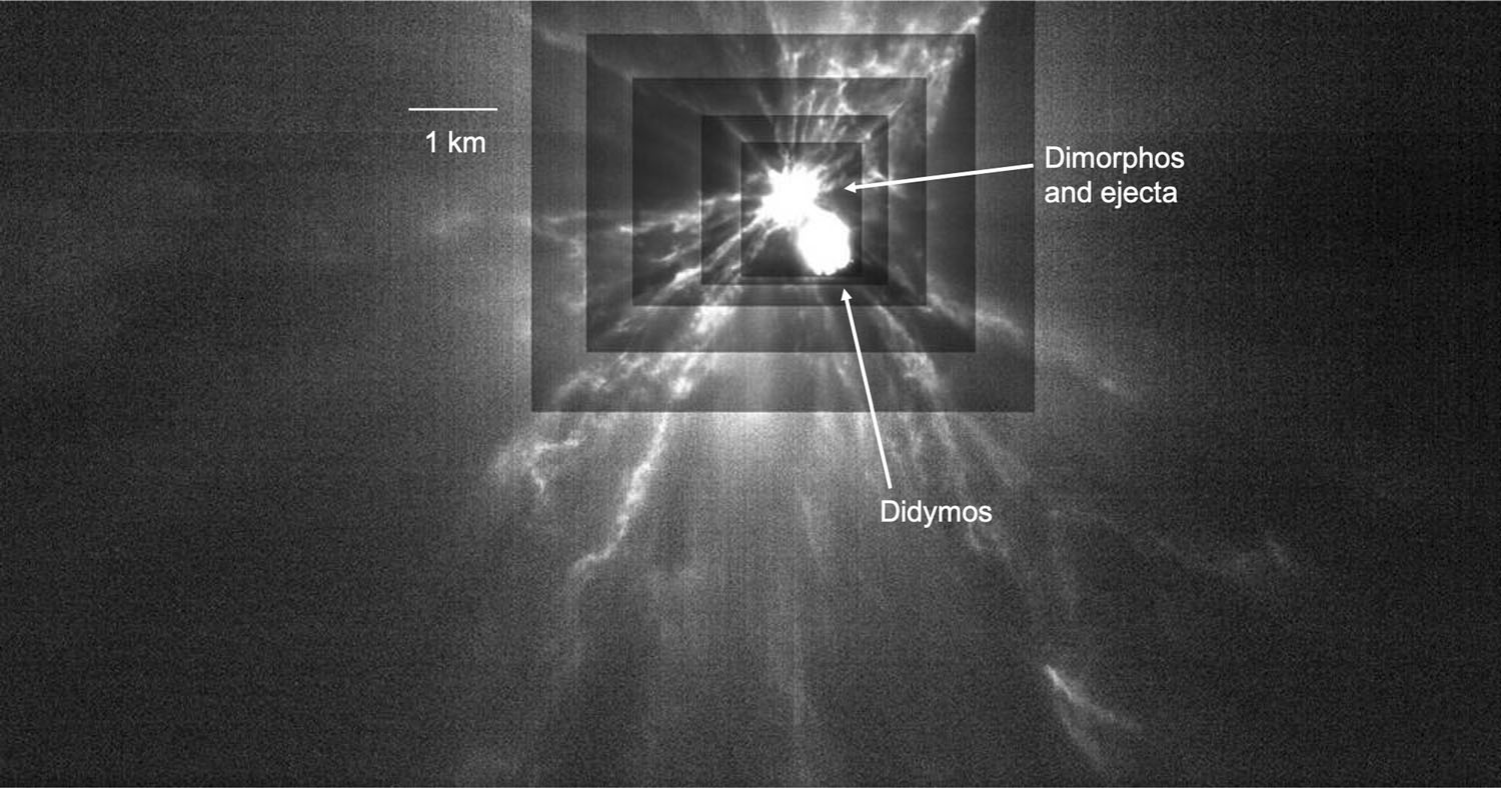
LICIACube image of the Didymos system after the DART impact. Filaments of ejecta are visible streaming away from the impact on Dimorphos. The contrast has been stretched in a differential manner across the image to make ejecta across the frame appear to be at the same brightness. The contrast is increased with each larger box. Arrows point to Didymos and Dimorphos, with the ejecta coming from the latter object. An estimated scalebar is included, based on the work of Daly et al.^15^. Credit: ASI/NASA/APL, adapted with permission.

DART was accompanied by the Light Italian CubeSat for Imaging of Asteroids (LICIACube), contributed by the Italian Space Agency. LICIACube studied the Didymos system with a color camera and a high-resolution monochrome camera in the minutes around the DART impact^4^. Filaments of rocky debris are seen stretching away from Dimorphos after the impact (Fig. 2^2,5^). Additional impact debris, or ejecta, is seen in Hubble Space Telescope (HST) images of the Didymos system shortly after the DART impact, and the ejecta has been followed by groundbased telescopes as it evolved into a tail^2^. Measurements of the ejecta can provide an independent estimate of the size of the push DART gave to Dimorphos, and continued monitoring can show the relative amounts of small particles and larger cobbles that were present in the debris. That knowledge can then be added to the measurements of the boulder distribution on Dimorphos to provide a fuller picture of the rocks that are present.

In 2024, the European Space Agency will launch the Hera mission, which will return to Didymos and Dimorphos in late 2026 for an extended stay^6^. Hera will be a second act to DART’s opening act, providing a comprehensive look at the system four years after DART’s impact, and making measurements that DART could not make like directly obtaining Dimorphos’ mass and imaging the portions of Didymos and Dimorphos that were unlit or out of sight at the time of DART and LICIACube’s arrival.

## Characterization

While we can be justifiably satisfied with the strides we have taken in mitigation studies and technology development with DART, less flashy efforts devoted to characterization will continue. Laboratory studies of meteorites, telescopic measurements of asteroids, and spacecraft missions show that the near-Earth objects (NEOs) span a wide variety of compositions, densities, and strengths^7^. Our ability to prepare for asteroid deflection efforts depends on characterizing possible impactors. These efforts will focus on both specific objects and on population studies, to let us better prepare for the most likely natures of yet-undiscovered objects.

An example of the former is the asteroid Apophis. Apophis is of particular interest because it comes exceedingly close to Earth in April 2029, passing only 31,000 km from the Earth’s surface—closer than the altitude of typical communications satellites. Apophis-sized objects (roughly 340 meters diameter) only come that close to Earth every 1000 years on average, and asteroid scientists meet regularly to discuss how best to learn about asteroids for planetary defense purposes from this approach^8^. We might anticipate an observing campaign around the time of Apophis’ close pass that is reminiscent of what has been organized for the DART impact.

The continuing efforts to search for NEOs will also help characterize the population. When the Rubin Observatory comes online next year, it is expected to greatly increase the rate of NEO discoveries and provide some compositional information by observing the colors of objects^9^. In addition to the search efforts for which it was designed, the NEO Surveyor project will also greatly benefit characterization efforts for the larger population of NEOs^10^. In particular, the ability of NEO Surveyor to measure the reflectivity of objects allows a rough composition to be inferred along with an improved estimate of the NEO population’s size distribution.

## Role of space missions in planetary defense

While DART was the first planetary defense mission to visit an asteroid, visits to asteroids by science missions have provided information that is critical for planetary defense characterization and mitigation planning. The samples returned by Hayabusa and Hayabusa2 and anticipated for OSIRIS-REx provide ground truth for remote compositional studies and give confidence in inferred compositions of Didymos and Dimorphos. Conversely, the unexpectedly blocky natures of the asteroids Bennu and Ryugu have led us to reconsider how we estimate surface roughnesses from telescopic data. OSIRIS-REx showed that Bennu is constantly emitting mm-cm-sized particles^11^, and the Japanese DESTINY + mission will visit an asteroid that is the parent of a meteor shower^12^. Understanding the way that NEOs lose mass and the dustiness of their environment, together with the ejecta studies from DART, will help us optimize the ways of safely interacting with asteroid surfaces if deflection is necessary.

OSIRIS-REx and Hayabusa2, which have already provided important information about NEOs via their prime missions, have extended missions that will give additional planetary defense insights. Hayabusa2 has a planned flyby with asteroid 2001 CC21 in 2026 and a rendezvous with asteroid 1998 KY26 in 2031. This second object is of particular interest since it is estimated to only be 30 m in diameter, roughly the same size as the Chelyabinsk impactor. It will be by far the smallest object visited by a spacecraft. Objects of this size are thought to hit the Earth about once a century, and we have little understanding of the nature of objects of this size. OSIRIS-REx has a planned rendezvous with Apophis in 2029, within days of Apophis’ close approach to Earth mentioned above.

## Future of mitigation missions after DART

The future of mitigation demonstrations is uncertain. We might expect, if only statistically, that deflection options can be demonstrated in non-emergency situations. There have been reports that the Chinese space agency will launch a kinetic impactor demonstration on the asteroid 2020 PN1 in 2025 or 2026, but few public details are available^13^. So-called slow push methods have yet to be tested. In these methods, a long-lasting but small force is applied to the asteroid in question to change its orbit rather than an impulsive force. An enhanced gravity tractor, an example of such a slow-push method, was proposed as part of NASA’s human exploration program in the last decade, but has not proceeded. The Planetary Science Decadal Survey included demonstrating a slow-push method as a candidate for the next mitigation test mission for NASA to fly, with a DART-like kinetic impactor at a smaller target with a faster closing speed as another candidate option^10^.

## International nature of planetary defense

While many endeavors in space science and exploration are single-nation affairs, planetary defense by its nature is an international issue. This international nature has been most obviously reflected in the DART mission by LICIACube^4^ and the follow-up Hera mission^6^, itself an effort by an international agency. Perhaps less obvious but as important to the success of DART was the contribution of time and effort by members of the Investigation Team all over the world, made possible because of the support of institutions all over the world. Similarly, investment in astronomy by nations like Chile and South Africa allowed critical measurements in support of the DART mission to be made.

At an intergovernmental level, international communication is fostered by the United Nations Office of Outer Space Affairs (UNOOSA). Of particular note is the Space Mission Planning Advisory Group (SMPAG), which brings together UN members and non-governmental entities and would serve as a means to assess options in case of an actual impact warning^14^. We must continue to work together as an international community to address planetary defense, and to continue to be transparent with the public whether we are livestreaming a spacecraft’s approach to an asteroid aiming to hit it, or doing more mundane work.

The bombardment history of the Earth, including last decade’s Chelyabinsk impact and the upcoming flyby of Apophis, demonstrate that even if we do not go to the NEOs, they may still come to us. The success of DART and the plans for advancing planetary defense in coming years should reassure the public that we are preparing for whatever may come our way.
